# Automatic segmentation of skin cells in multiphoton data using multi-stage merging

**DOI:** 10.1038/s41598-021-93682-y

**Published:** 2021-07-15

**Authors:** Philipp Prinke, Jens Haueisen, Sascha Klee, Muhammad Qurhanul Rizqie, Eko Supriyanto, Karsten König, Hans Georg Breunig, Łukasz Piątek

**Affiliations:** 1 Institute for Biomedical Engineering and Informatics, Technische Universität Ilmenau, 98693 Ilmenau, Germany; 2 Division Biostatistics and Data Science, Department of General Health Studies, Karl Landsteiner University of Health Sciences, Dr. Karl-Dorrek-Straße 30, 3500 Krems, Austria; 3 Informatics Engineering Program, Universitas Sriwijaya, Palembang, South Sumatera Indonesia; 4 IJN-UTM Cardiovascular Engineering Centre, Universiti Teknologi Malaysia, Johor Bahru, Johor, Malaysia; 5 Department of Biophotonics and Laser Technology, Saarland University, Campus A5.1, 66123 Saarbrücken, Germany; 6 JenLab GmbH, Johann-Hittorf-Straße 8, 12489 Berlin, Germany; 7 Department of Artificial Intelligence, University of Information Technology and Management, H. Sucharskiego 2 Str, 35-225 Rzeszów, Poland

**Keywords:** Image processing, Classification and taxonomy

## Abstract

We propose a novel automatic segmentation algorithm that separates the components of human skin cells from the rest of the tissue in fluorescence data of three-dimensional scans using non-invasive multiphoton tomography. The algorithm encompasses a multi-stage merging on preprocessed superpixel images to ensure independence from a single empirical global threshold. This leads to a high robustness of the segmentation considering the depth-dependent data characteristics, which include variable contrasts and cell sizes. The subsequent classification of cell cytoplasm and nuclei are based on a cell model described by a set of four features. Two novel features, a relationship between outer cell and inner nucleus (OCIN) and a stability index, were derived. The OCIN feature describes the topology of the model, while the stability index indicates segment quality in the multi-stage merging process. These two new features, combined with the local gradient magnitude and compactness, are used for the model-based fuzzy evaluation of the cell segments. We exemplify our approach on an image stack with 200 × 200 × 100  μm^3^, including the skin layers of the stratum spinosum and the stratum basale of a healthy volunteer. Our image processing pipeline contributes to the fully automated classification of human skin cells in multiphoton data and provides a basis for the detection of skin cancer using non-invasive optical biopsy.

## Introduction

Malignant melanoma is a skin cancer that develops from melanocytes, which are pigment-containing cells found in the basal layer. The melanoma grows rapidly and easily metastasizes^1^. It is highly lethal in cases where it is not diagnosed early. Early diagnosis allows a five-year relative survival rate of up to 98%, as stated by the American Cancer Society concerning the USA^2^. Thus, accurate and early diagnosis methods are needed.

Considering a typical size of a skin cell in the basal layer of about 10 μm, a resolution of 1 μm or better is required for the representation of subcellular details. Optical high-resolution non-invasive imaging methods with a morphological-functional basis, such as in vivo multiphoton tomography (MPT), are suitable for this purpose^3^.

Subcellular morphological details of tissue can be displayed without exogenous marker substances (label-free) based on molecules themselves. In particular, the mechanisms of two-photon-excited fluorescence (TPEF)^4^ and second harmonic generation (SHG)^5^ are used. Additionally, complementary detection of the fluorescence in a time-resolved fashion provides access to fluorescence lifetime imaging (FLIM)—a further contrast method with chemical selectivity^3,6–8^. The combination of chemical-selective imaging and subcellular spatial resolution gives insight into the metabolism of cells and thus provides access to functional imaging^9–11^. Based on these methods, a new clinical application has been established, namely, high-resolution non-invasive and non-destructive imaging of living tissue—known as nonlinear "optical biopsy"^12–14^. This provides the basis for an early diagnosis at the cellular level or a course assessment in a physiological environment.

The research field of detecting malignant melanomas by means of MPT is still in its infancy and, in addition to the validation of suitable features, requires the development of image processing algorithms for the automatic classification of tissue structures.

### Related work

The segmentation of skin cells in multiphoton images has already been discussed in a number of publications. Walsh and Skala^15^ proposed a multi-threshold technique to differentiate between cell nuclei, cells, and background in skin-like TPEF data of human breast cancer cells. The cell components are separated from the background using a four-component fitting model. This method requires a specific grey value distribution and, thus, a clear delimitation of the cell components without uneven illumination drift and a high nucleus contrast. The maximum permissible core size is limited by a manually set threshold. Decencière et al.^16^ use a tissue segmentation method based on mathematical morphology to analyze in vivo skin data obtained by multiphoton tomography. After a morphological closing operation to remove objects below a manually defined minimum size and a reconstruction by erosion, a filter operation is carried out by means of a threshold value regarding the noise level. Another approach by Decencière et al.^17^ uses SHG in addition to the multiphoton data for a rough segmentation of skin layers by demarcation of the three main compartments named: coupling medium on the skin, epidermis, and dermis. Thus, a watershed transformation is performed on markers based on empirically chosen thresholds. The final fusion is done by a graph cut algorithm. Wu et al.^18^ also utilize combined SHG and TPEF data for high contrast of cells and fewer artefacts in rabbit elastic cartilage two-photon images to segment outer cell boundaries. Primitives based on superpixels are constructed by involving a distance metric in combination with spatial features, as well as CIE Lab color space values and phase congruency. The subsequent watershed transformation based on the primitives yields the final segmentation.

The watershed transformation to segment rough cell boundaries in reflectance confocal microscopy is also performed by Chen et al.^19^. Based on these boundaries, as an initialization of a level set model, the recognition of nuclei and cytoplasm in two-photon data is realized. Bates et al.^20^ used a machine learning algorithm for segmentation of vasculature. Supervoxels describe an initial image segmentation as input to feature extraction by a random forest classifier. The subsequent regularization is done by Markov random fields. An application of the algorithm in cell detection is conceivable by using appropriate training data. Another algorithm based on a random forest classifier was proposed by Machairas et al.^21^. The authors compare the advantages of adaptive features by superpixel area and rectangular regions of sliding window approaches for the segmentation of melanocytes images. The extraction of superpixels is done by the watershed transformation. The features of those regions are used as inputs for a random forest classification. The classification fails where contours are blurred. Due to the limited database, a review of the generality of the solution is required. Hu et al.^22^ used FLIM images for a multiresolution community detection method based on graph partitioning theory. The manual entry of the network resolution determines the size of the recognizable segments, where a low network resolution results in large segments and vice versa.

In recent years, the segmentation and classification of cell images based on reflectance confocal microscopy (RCM) data has received more research attention. Due to the similarity of the characteristics of RCM and MPT data, these methods are partly adaptable to MPT images. Arce et al.^23^ used a mixture of Gaussian-based adaptive thresholds on pre-processed sharpened images to extract cell boundaries of single cells on a homogeneous background. A model-based nucleus segmentation algorithm was proposed by Arslan et al.^24^. The nuclei boundaries are described in their algorithm by four types of primitives with different orientations, which are concatenated by an attributed relational graph. After searching for predefined structural patterns in the graph, a region growing technique is used to find the final contours. Restrictions are given especially by the fixed parameterization (e.g., the thresholds for primitive length, image gradients, and compactness). Maška et al.^25^ used a shape-tracking algorithm for cells based on the Chan–Vese model^26^ for initial segmentation and a fast level set and graph-cut framework for tracking. A basic prerequisite for the segmentation step is a clear distinction between the individual cells. A high manual effort is required to separate the cell clusters for subsequent tracking. Another approach of cell tracking was proposed by Tarnawski et al.^27^. The initial segmentation is done by the H-minima transform in combination with the watershed transformation and ellipse fitting on data with a homogeneous background. After segmentation, a modified multiphase active contour is used for tracking. Chen et al.^28^ used a supervised learning-based template-matching approach for nuclei segmentation. For this purpose, a statistical model for texture and shape variations is generated by principal component analysis on ground truth data. The cross-correlation with the template ensures the nuclei recognition on nearly homogeneous backgrounds in the evaluation step. Another approach for cell segmentation has been described by Dimopoulus et al.^29^. Starting from seed-points derived by the Hough transformation in cell segments, a cross-correlation of membrane patterns to patterns from reference is determined. The tracing of the cell boundary is done by a graph-cut algorithm. Restrictions result from the choice of parameters, such as cell size and average membrane patterns, in terms of intensity and position, especially in combination with irregular cell boundaries and non-uniform patterns.

Here we introduce publications that have discussed cell segmentation on other modalities. Wienert et al.^30^ used a minimum-model approach for nucleus detection in haematoxylin and eosin staining data. The segmentation was done by a combination of closed image contours recognition and maximization of mean gradient and gradient fit parameters. Several fixed parameters restrict the extractable object size. Due to the maximization of the mean gradient, it is a problem to detect weak nuclei contours without introducing further artefacts. The nuclei and cytoplasm were segmented by Lu et al.^31^ using an unsupervised classification for initial cell clumps and the maximally stable extremal regions (MSER) algorithm for nuclei detection. The final segmentation was achieved by using a joint level set optimization with respect to initial detection. Overlapping objects or poor contrasts mean that the affected objects are not recognized. A semi-automatic approach for segmentation and classification of nuclei and cytoplasm has been proposed by Chen et al.^32^. Contours were extracted using an adaptive threshold and maximum grey-level gradient difference for cells and nuclei, respectively. Afterwards, the contours of multiple cells were adjusted by a manual random walk detector. After computation of morphometric and textual features (depending on RGB data), a support vector machine was used to classify different types of cells in images with significant borders and low noise.

Dima et al.^33^ used a bivariate similarity index metric for a comparison of cell estimations by nine segmentation approaches on fluorescence microscopy data. As a result, k-means clustering methods with multiple clusters were shown to be superior to the segmentation results of threshold-based segmentation techniques. Meijering^34^ showed a variety of solutions for cell segmentation based on fluorescence microscopy deploying intensity thresholding, feature detection, morphological filtering, region accumulation, and deformable model fitting. Dima et al.^33^ and Meijering^34^ also include recent reviews of cell segmentation approaches.

The methods presented in the above paragraphs have limitations with respect to their use on low-contrast data with high variance in cell shape and size and irregularity of cell boundaries. These limitations have been partially mitigated by processing additional modalities. For our application, only autofluorescence data from the MPT are available. Although machine learning algorithms have been used successfully to extract cell features in different modalities^20,21,28,29,35^, they require a sufficiently large amount of training data to ensure a generalized solution for segmentation due to the high variability of skin cells in shape and appearance. For MPT, currently, only low amounts of data are available.

### Contributions

It is noticeable that many authors have used manual thresholds for minimal contrasts, but also for cell sizes. The natural tissue is characterized by decreasing cell sizes toward deeper skin layers as well as acquisition-induced intensity fluctuation of the cell boundary and shading effects in lateral border areas of the image. A discontinuity of the cell boundary is caused partly by low contrast, but also by the appearance of clumps of cells and of background artefacts. For these reasons, we propose an algorithm that avoids manual restrictive thresholds. Our approach consists of three main modules for the automatic segmentation of skin cells in multiphoton data. The pre-processing (module 1) involves the preparation of the data material regarding a robust segmentation by a watershed transformation with subsequent multi-stage merging (module 2). The semantic assignment (module 3) includes the extraction and fuzzy-like evaluation of specific features to calculate a final characteristic for all cell segments.

## Materials and methods

### Materials

Multiphoton tomography is a non-invasive technique^4^ that allows in vivo skin assessment. The skin contains a variety of endogenous fluorophores (such as Nicotinamide adenine dinucleotide phosphate in reduced form (NAD(P)H), melanin, elastin, and collagen)^9^. These natural fluorescence markers generate a signal by means of MPT, which represents morphological cell structures. A significant signal is produced by mitochondria in the cytoplasm of cells mainly by the presence of NAD(P)H. In contrast, the cell nuclei are low in signal due to the absence of NAD(P)H and other fluorophores^36^. The cellular autofluorescence is mainly based on the NAD(P)H fluorescence^37^.

**Figure 1:**
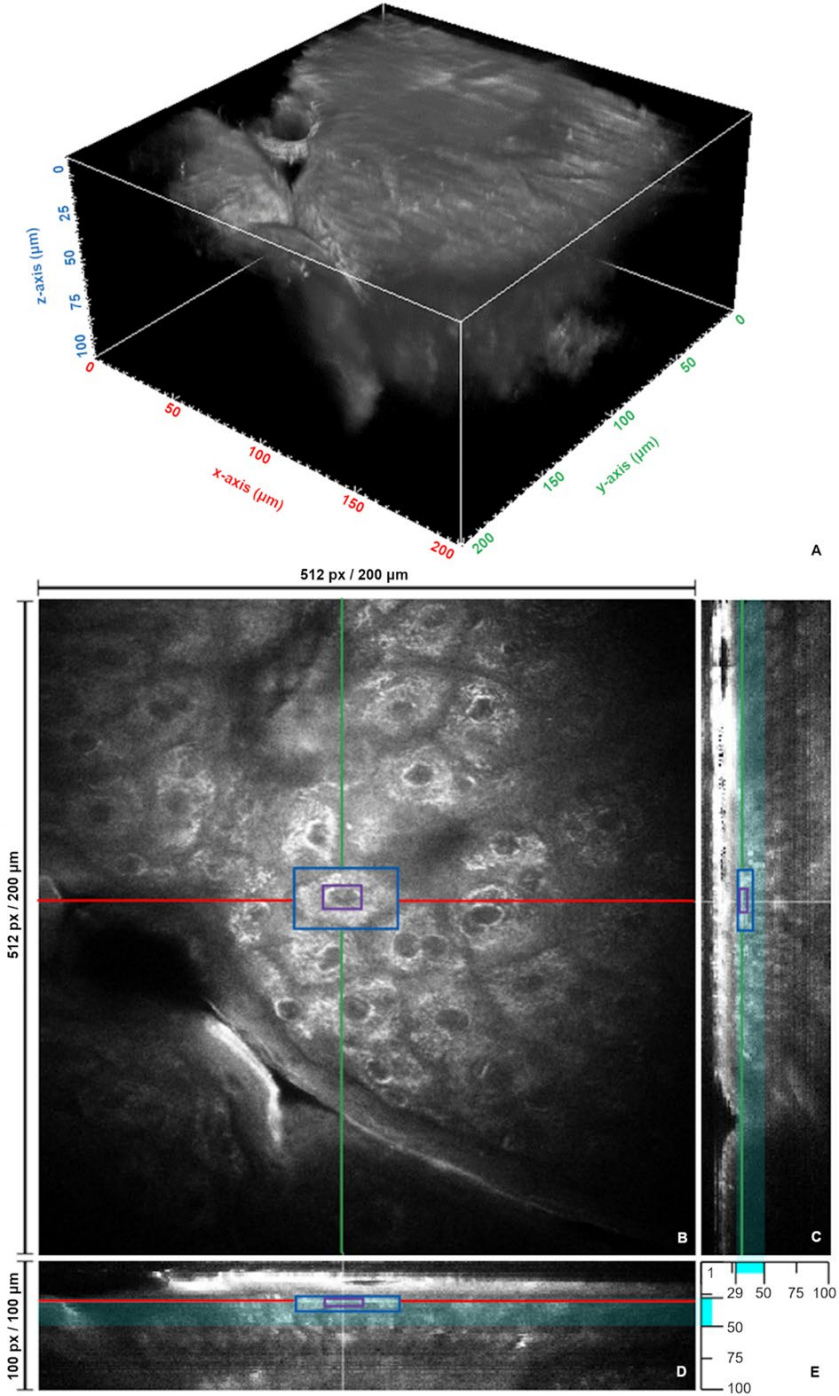
Image data. (**A**) 3D representation of the image stack with a size of 200 × 200 × 100 μm^3^; (**B**) X–Y-plane in depth of 31 μm with cell bounding box (blue), nucleus bounding box (violet), intersection line of Y–Z-plane in (**C**) (green), and intersection line of X–Z-plane in (**D**) (red); (**C**) cross-section in Y–Z-direction; (**D**) cross-section in X–Z-direction; and (**E**) visualization of the considered cell-containing Z-layer interval in X–Z- and Y–Z-planes (cyan bars) with upper (29 μm) and lower (50 μm) boundaries.

The autofluorescence data for this publication were generated with an MPT*flex*™ (JenLab GmbH, Berlin, Germany), a CE-certified medical tomograph with modules for two-photon TPEF, SHG, and FLIM signal recording in vivo^38^. Measurements were approved by the Ethics Committee of the Medical Association of the Free State of Thuringia and informed consent was obtained. All methods were performed in accordance with the relevant guidelines and regulations. The presented algorithm takes only the TPEF data into account. These data are organized in a two-dimensional image stack (Fig. 1). The lateral image resolution is 512 × 512 pixels with a sampling step size of 0.39 μm, which results in an imaged region of 200 × 200 μm^2^, taking 6445.06 ms for acquisition. The step size in axial direction is 1 μm, with the maximum layer depth at 100 μm. The grey scale values of the resulting 512 × 512 × 100 voxels indicate the number of photons counted by the detector.

While the first layers are mainly characterized by structures without distinctive cell-nucleus networks, cells increasingly appear in the following layers (Fig. 1). With increasing depth, the cells become smaller. Their size depends on the cell type. Whereas coreless corneocytes are present in the upper epidermis layer (stratum corneum), the cell density in the stratum granulosum and in the stratum spinosum increases steadily down to the stratum basale with a simultaneous decrease of the cell volume. Keratinocytes have the smallest cell volume with comparably large cell nuclei in the stratum basale as the deepest layer of epidermis^39^. The size of the skin cells depends also on other factors, such as gender, skin area, age, and hydration status of the skin cells. The cell volume can increase by a factor of two with increasing water binding^40,41^. Due to the variable depth-dependent image data characteristics, a sufficiently robust methodology is required.

Figure 1B shows a multiphoton image from the stratum spinosum at a depth of 31 μm. The cell in the blue bounding box measures 29.5 × 16.9 × 7.0 μm^3^. The autofluorescence of the cytoplasm, the lower intensity of the nuclear structure, and the intercellular matrix as a cell separation are clearly visible. In general, the data have a low contrast, a granular background distribution, and a signal drop toward the edge of the image.

A reference segmentation is used to validate the results of our proposed algorithm for automatic segmentation of skin cells using multi-stage merging. The reference segmentation was done by two experts who marked pixel-precisely the contours of the cells and nuclei on 14 slices of a single 2D image data stack. The experts marked up to 797 cells and nuclei with a border color scheme to be able to delineate uncertain object boundaries by specific coloring. According to these experts, 179 of the 797 cells were considered uncertain. In the following analysis, we use only the opinion from expert one as ground truth, because expert two used a more conservative approach and labelled fewer cells compared to expert one (774 vs. 484 cells labelled). Only 23 out of 797 cells were labelled by expert two but not by expert one. By using 14 layers of different depths (especially 29–40, 45, 50 μm) see Fig. 1 for localization in the overall image stack), a cross-section of the cell characteristics in terms of shape and size is given. This reference segmentation is called ground truth, $GT$, in the following discussion. The validation is done using the Dice coefficient, $DC$^42^, which determines the similarity between $GT$ and the automatic segmentation result, and ranges between 0 and 1:

$$DC = \frac{{2 \cdot TP}}{{2 \cdot TP + FP + FN}},$$

where $TP$ means true positive, $FP$ false positive, and $FN$ false negative.

### Methods

For developing a segmentation pipeline for skin cells in multiphoton data, the following requirements are given due to the data properties:

- the wide variety of object sizes requires small sliding windows,
- cell and nuclei appearance in terms of topology and grey value distribution should be considered, and
- varying local grey scale ratios and image edge-related intensity drops should be considered.

**Figure 2:**
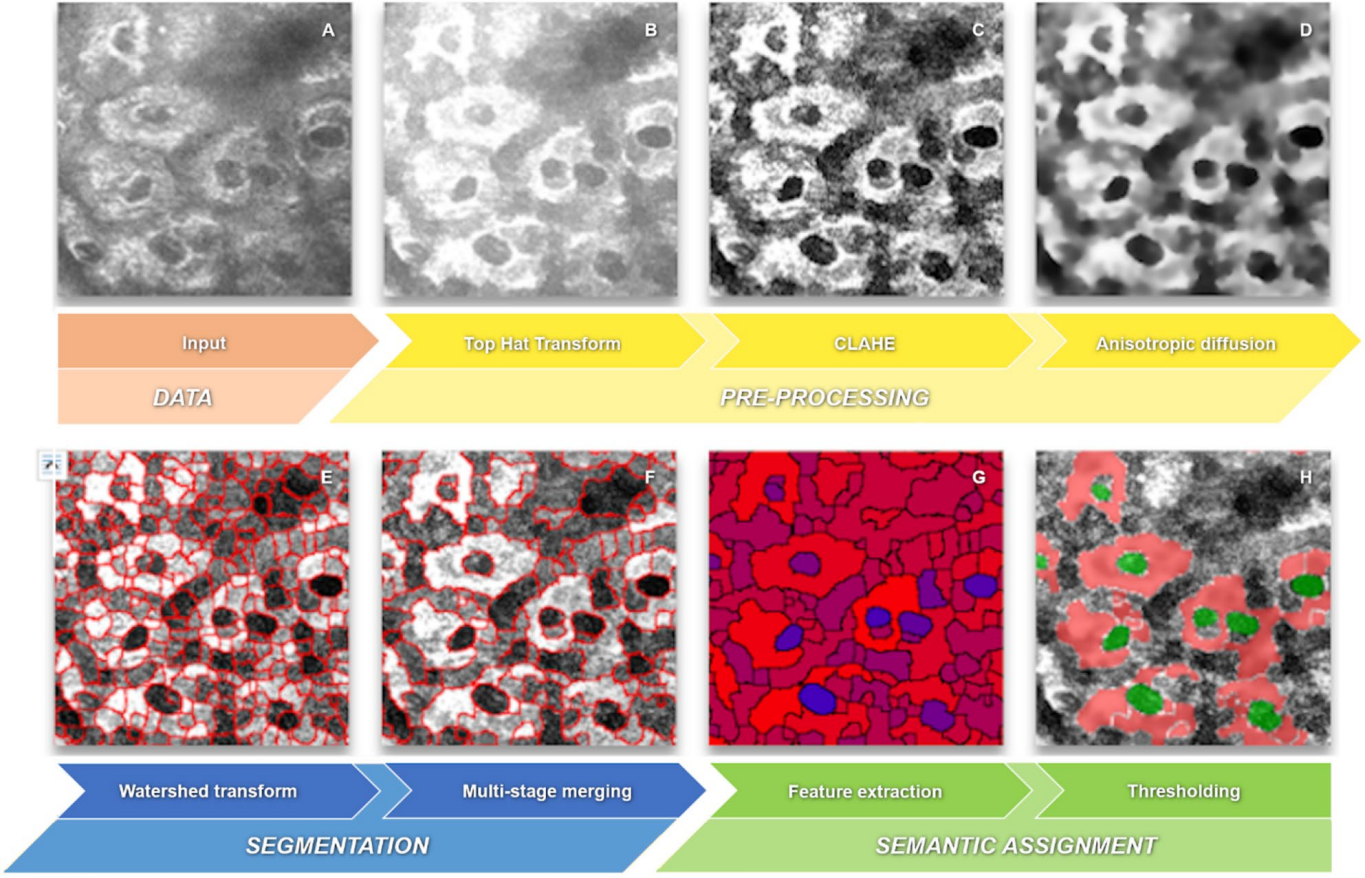
Processing pipeline. Example multiphoton input image from slice 30 (**A**) for illustrating the automatic segmentation of skin cells (**H**, cytoplasm (red), nuclei (green)) in subsequent steps: plateauing by Top Hat transform (**B**), contrast enhancement by CLAHE (**C**), edge preserving filtering by anisotropic diffusion (**D**), segmentation (boundaries are red) by Watershed transform (**E**), fusion of over-segmented regions to semantic regions (boundaries are red) by multi-stage merging (**F**), feature extraction using the vector 2-norm (**G**) of the extracted suitable features with respect to model features (minimum vector range (blue) corresponds to greatest similarity to the nucleus model) and subsequent final thresholding (**H**).

The fully automated segmentation with feature-based fuzzy object classification includes the following three modules (Fig. 2): *pre-processing*, *segmentation*, and *semantic assignment*.

#### Pre-processing

The pre-processing module prepares the TPEF data for subsequent segmentation in module 2. The image regions of the cells appear highly inhomogeneous due to the arrangement of fluorescents in the cytoplasm. The morphological method of *top hat transformation*^43^ with the combination of white top hat ${T}_{w}$

$$T_{w} \left( {I,H} \right) = I - I \circ H$$

and black top hat transformation ${T}_{b}$

$$T_{b} \left( {I,H} \right) = I \cdot H - I$$

was used to generate homogeneous regions from areas with high image frequencies, where $I$ is the original TPEF image, $H$ is the circular structuring element with a diameter of 11, $\circ$ denotes morphological opening, and · represents morphological closing. The white top hat transformation emphasizes smaller image areas with high intensities in terms of the size of the structuring element, while the black top hat emphasizes small image regions with low intensities. The sum of white and black top hat corresponds to the arithmetic difference between closure and opening of the image and is suitable for increasing the contrast. Plateau formation occurs in $I_{{TH}}$ when the original TPEF image is added to the contrast enhancement image:

$$I_{{TH}} = I + ~T_{w} \left( I \right)~ + ~T_{b} \left( I \right).$$

This transformation highlights contiguous high-intensity image areas that are a priori likely to be the cytoplasm surrounding cell nuclei (Fig. 2B).

Since the result of the top-hat transformation still has a low contrast and an edge-increasing signal drop (the so-called shading), a subsequent contrast manipulation was implemented to exploit the entire tonal range. The method of *contrast-limited adaptive histogram equalization* (CLAHE)^44^ was used on image ${I}_{TH}$ to get the contrast enhancement result ${I}_{CE}$. The adjusted pixel value in ${I}_{CE}$ results directly from the cumulative distribution function (CDF). The CDF corresponds to the cumulative histogram, which was calculated from the grey value distribution of the region of interest (ROI). The ROIs result from the size of the kernel and the principle of sliding windows. The contrast limitation for suppressing the noise amplification was realized by clipping the histogram amplitude before calculating the CDF. This limits the maximum number of individual pixel values in each ROI and, thus, the slope of the CDF. Pixel elements above the clipped amplitude threshold are equally distributed among the histogram bins. The result is an increase in local contrast without amplification of local noise (Fig. 2C). Another effect of using the CLAHE in ROIs is the realization of the shading correction.

The contrast enhancement is followed by the filtering of the image noise. Edge-preserving filtering is used according to the *anisotropic diffusion* principle by Perona and Malik^45^. This corresponds to an iterative smoothing filter with a restriction of smoothing in the direction of the gradient in the gradient image $G$. Thus, smoothing of the image $I={I}_{CE}$ is only done orthogonally to the edge-gradient direction, while smoothing toward the direction of the gradient is avoided. The diffusion equation

$$I_{{i,j}}^{'} = I_{{i,j}} + \frac{1}{\eta }div\left( {d\left( {\left| {\nabla {\text{G}}} \right|} \right)\nabla I_{{i,j}} } \right)$$

calculates an anisotropic filtered image ${I}_{i,j}^{\text{'}}$ with the diffusion coefficient

$$d\left( {\left| {\nabla {\text{G}}} \right|} \right) = \frac{1}{{1 + \left( {\frac{{\left| {\nabla {\text{G}}} \right|}}{K}} \right)^{2} }}~,$$

with $\eta = 8$ for the vicinity of a pixel with index $i,j$ in the eight cardinal directions, $\nabla$ denotes the gradient, $div\left( \cdots \right)$ the divergence operator, and the constant $K$ regulates the sensitivity to the edges. Let $d_{{i + 1,j,t}} ,d_{{i - 1,j,t}} ,d_{{i,j + 1,t}} ,d_{{i,j - 1,t}}$ be the diffusion coefficients in northern, southern, eastern, and western directions and $d_{{i + 1,j + 1,t}} ,d_{{i - 1,j + 1,t}} ,d_{{i - 1,j - 1,t}} ,d_{{i + 1,j - 1,t}}$ in northeast, southeast, southwest, and northwest directions with respect to the pixel indices $i,j$, then the anisotropic diffusion according to Perona and Malik can be defined discretely as follows:

$$\frac{{dI_{{i,j}} }}{{dt}} = \frac{\lambda }{8}\left\{ {\begin{array}{*{20}c} {d_{{i + 1,j,t}} \left[ {I_{{i + 1,j}} - I_{{i,j}} } \right] + d_{{i - 1,j,t}} \left[ {I_{{i - 1,j}} - I_{{i,j}} } \right]} \\ { + d_{{i,j + 1,t}} \left[ {I_{{i,j + 1}} - I_{{i,j}} } \right] + d_{{i,j - 1,t}} \left[ {I_{{i,j - 1}} - I_{{i,j}} } \right]} \\ {~ + d_{{i + 1,j + 1,t}} \left[ {I_{{i + 1,j + 1}} - I_{{i,j}} } \right] + d_{{i - 1,j + 1,t}} \left[ {I_{{i - 1,j + 1}} - I_{{i,j}} } \right]} \\ { + d_{{i - 1,j - 1,t}} \left[ {I_{{i - 1,j - 1}} - I_{{i,j}} } \right] + d_{{i + 1,j - 1,t}} \left[ {I_{{i + 1,j - 1}} - I_{{i,j}} } \right]} \\ \end{array} } \right\},$$

with the parameter $\lambda$ to control the smoothing effect and $t$ as the number of iterations. The filtered image ${I}_{AD}$ (Fig. 2D) results from

$$I_{{AD}} = I_{{i,j}}^{{t + 1}} = I_{{i,j}}^{t} + \frac{{dI_{{i,j}} }}{{dt}}\;with\;I_{{i,j}}^{0} = I_{{CE}} .$$

#### Segmentation

The prepared image of the pre-processing module is transferred to the segmentation module for grouping the individual iconic pixels into semantic pixel clusters. The clusters are extracted by the *watershed transform* algorithm, as proposed by Meyer and Beucher^46^.

The watershed transformation is performed on the gradient magnitude image ${G}_{AD}$

$$G_{{AD}} = \sqrt {D_{x}^{2} + D_{y}^{2} }$$

of the pre-processing result $I_{{AD}}$ to provide segment boundaries on the object edges. The partial orthogonal derivatives

$$D_{x} = I_{{AD}} *H_{{Sx}} \;with\;H_{{Sx}} = \left[ {\begin{array}{*{20}c} { - 1} & 0 & 1 \\ { - 2} & 0 & 2 \\ { - 1} & 0 & 1 \\ \end{array} } \right]~$$

and

$$D_{y} = I_{{AD}} *H_{{Sy}} \;with\;H_{{Sy}} = \left[ {\begin{array}{*{20}c} { - 1} & { - 2} & { - 1} \\ 0 & 0 & 0 \\ 1 & 2 & 1 \\ \end{array} } \right]$$

are determined by the convolution kernels $H_{{Sx}}$ and $H_{{Sy}}$ of the Sobel operator. The watershed transformation results in an over-segmented image. Based on the data properties, it is probable that the semantic objects, such as cells or cell nuclei, are divided into multiple segments. However, an undesired union of two semantic objects can per se be excluded if they are separated by a grey scale gradient. The degree of over-segmentation is slightly mitigated by convolution of the gradient magnitude image $G_{{AD}}$ with a Gaussian filter

$$g\left( {x,y} \right) = \frac{1}{{2\pi \sigma ^{2} }}e^{{ - \frac{{x^{2} + y^{2} }}{{2\sigma ^{2} }}}} ~,$$

where $x$ is the distance to the origin of the kernel in the horizontal direction, $y$ the distance in the vertical direction, and $\sigma$ is the standard deviation of the Gaussian distribution. The result of the watershed transformation $I_{{WS}}$ contains semantically correct segments in an over-segmented image (Fig. 2E) with the segment boundaries on the edge structures of the input image $I_{{AD}}$.

To assign only one segment to each semantic object, a newly developed merging strategy has been implemented: the approach of *multi-stage merging* on multiphoton data.

The proposed multi-stage merging is based on the over-segmentation result by the watershed transformation. These segments, as a group of coherent grey level pixels, act as superpixels^47^. Thus, the merging step is based on meaningful regions instead of single pixels. This leads to a reduction of the input entities for the subsequent multi-stage merging algorithm. The feature associated with a superpixel $s$ is the average intensity $\bar{I}_{s}$ of $s$ with $s = 0..\left( {k - 1} \right)$, where $k$ is the number of superpixels after watershed transformation.

Based on the superpixels, a second flooding process is performed (Algorithm 1), starting from the superpixel with the lowest intensity and moving in ascending order. Adjacent superpixels will be merged if their absolute difference of intensities $d = \left| {\bar{I}_{s} - \bar{I}_{n} } \right| \le T_{m}$ is smaller than a threshold $T_{m}$ for the merging step with $n = 0..\left( {k - 1} \right)$, $n \ne s$ and $k$ is the number of superpixels. $T_{m}$ specifies the range of the maximum possible quantization level. The new feature value for such a merged superpixel is obtained by the mean of all intensities from a single superpixel before merging. The flooding process ends when all the intensities of adjacent superpixels do not allow further union with respect to the selected threshold.
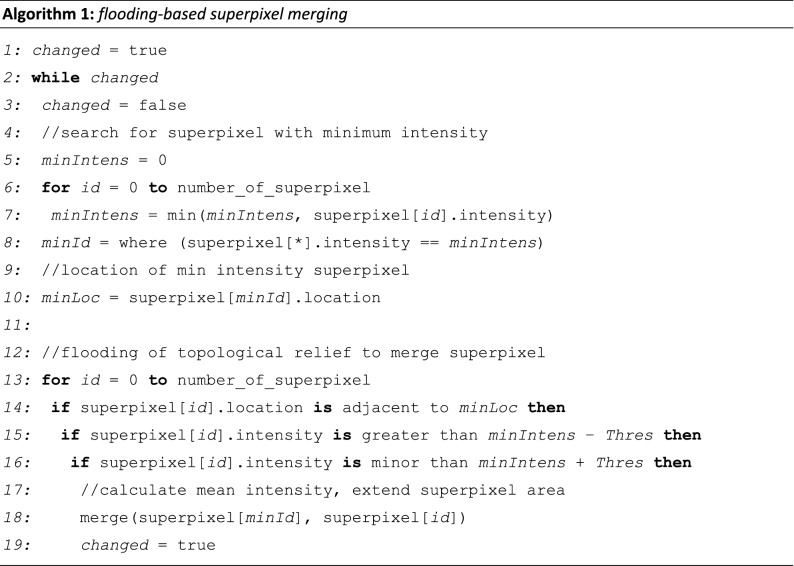


**Figure 3:**
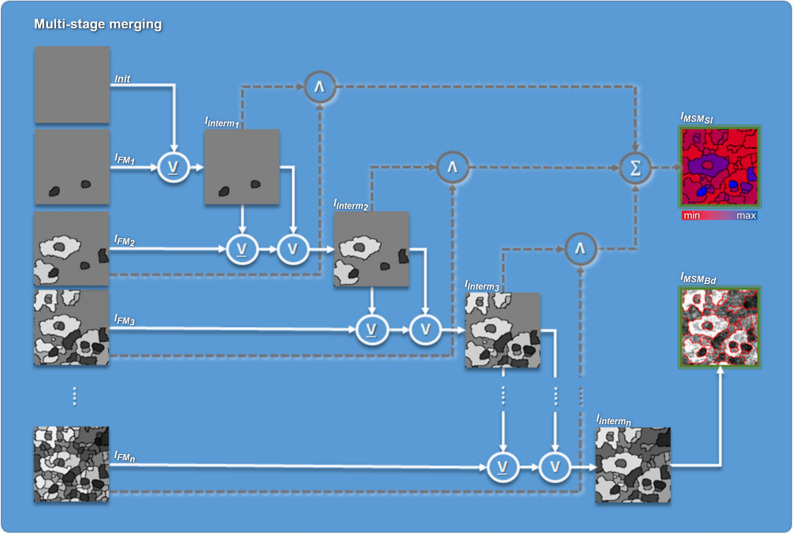
Multi-stage merging. Iterative process of merging using logical operation exclusive disjunction (⊻) and disjunction (∨) of initial segments $\left( {Init,I_{{FM_{1}}} ..I_{{FM_{n} }} } \right)$ with $n = 7$ and intermediate results $\left( {I_{{interm_{1} ~}} ..~I_{{interm_{{n - 1}} ~}} } \right)$ to extract final segments $\left( {I_{{interm_{n}}} } \right)$, augmentation of original image with segment boundaries (red outlines in $I_{{MSM_{{Bd}}}}$), and determination of stability feature ($I_{{MSM_{{SI}}}}$, indicates low (red) and high (blue) stability index) by overall summation of iterative conjunction (∧) of binarized segments.

Since the choice of the threshold $T_{m}$ affects the quality of the result considerably, the assumption of a single subjective value is critical due to the data variability described above. The manual selection of the most appropriate thresholds can be eliminated. Several thresholds, $T_{{m_{i} }} = 10 \cdot i$ with $i = 1..n$, are used. These threshold values should provide an equidistant coverage of the gradient magnitude range. For our specific dataset, we use $n~ = ~7$. Due to multiple thresholds, a merging step is required. This proposed multi-stage merging uses the results $I_{{FM_{i} }}$ of several simultaneous flooding cycles with various thresholds $T_{m}$ as input. The scheme of multi-stage merging is shown in Fig. 3.

The implementation of the scheme is realized as follows. Objects with maximum contrast are extracted first by merging the superpixels using high thresholds for $T_{m}$. Subsequently, all $T_{{m_{i} }}$ with $i = 1..n$ are used for merging. As the threshold decreases, less significant, lower-contrast objects are detected. A side effect is that the previously extracted objects split into multiple sub-objects. To prevent decomposition of the significant objects, only new objects in the previously masked background area are allowed. The background is defined as the largest contiguous segment after merging the superpixels and conceptually includes the lower-contrast segments with respect to the current threshold.

The iterative implementation can be done efficiently by means of logical operators. While exclusive disjunction prevents the disintegration of previously extracted objects, the disjunction inherits newly occurring objects from the initial images $I_{{FM_{i} }}$ into the current intermediate solution step $I_{{interm_{i} }}$ (Fig. 3):

$$I_{{interm_{i} ~}} = \left\{ {\begin{array}{*{20}c} {I_{{FM_{i} ~}} ,} & {if\;~i = 1;} \\ {\left( {I_{{interm_{{i - 1}} ~}} \underline{ \vee } ~I_{{FM_{i} }} } \right) \vee I_{{interm_{{i - 1}} ~}} ,} & {otherwise;~} \\ \end{array} } \right.$$

where $$\underset{\raise0.3em\hbox{$\smash{\scriptscriptstyle-}$}}{ \vee }$$ denotes the exclusive disjunction, $$\vee$$ denotes the disjunction, and $$I_{{FM_{i} }}$$ are the images calculated by flooding-based merging of superpixels (Algorithm 1) using the $$n$$ thresholds $$T_{{m_{i} }}$$ for $$i = 1..n$$. The intermediate solution subsequently serves as input data for the next iteration of multi-stage merging. The final solution $$I_{{interm_{n} }}$$ (Fig. 3) represents a segmented image $$I_{{MSM}}$$ in which contiguous high-contrast objects, as well as the less significant objects, are represented by their mean intensity. The object boundaries can be finally augmented in the original data (Figs. 2F, 3$$I_{{MSM_{{Bd}} }}$$).

We derive a parameter of *stability index* $S$ (Fig. 3$I_{{MSM_{{SI}} }}$)

$$S = \mathop \sum \limits_{{i = 1}}^{{n - 1}} \left\| {I_{{interm_{i} }} ~\Lambda ~I_{{FM_{{i + 1}} }} } \right\|,$$

where $\left\| X \right\|$ denotes the norm for extraction of a binary mask $mask_{{bin}}$ for expression $X$:

$$mask_{{bin}} \left( X \right) = \left\{ {\begin{array}{*{20}c} {0,~} & {if\;X = 0.} \\ {1,} & {otherwise.~} \\ \end{array} } \right.$$

The stability index $S$ considers here the ${\text{n}} = 7$ processing stages. For this purpose, the sum over all conjunctions between the intermediate results $I_{{interm_{i} }}$ and the $T_{m}$-merged images from the next iterations $~I_{{FM_{{i + 1}} }}$ is used. A high stability index value is achieved for high-contrast segments. We use this parameter also to map the previously unclassified segments to an anatomical class, as shown below.

#### Semantic assignment

**Figure 4:**
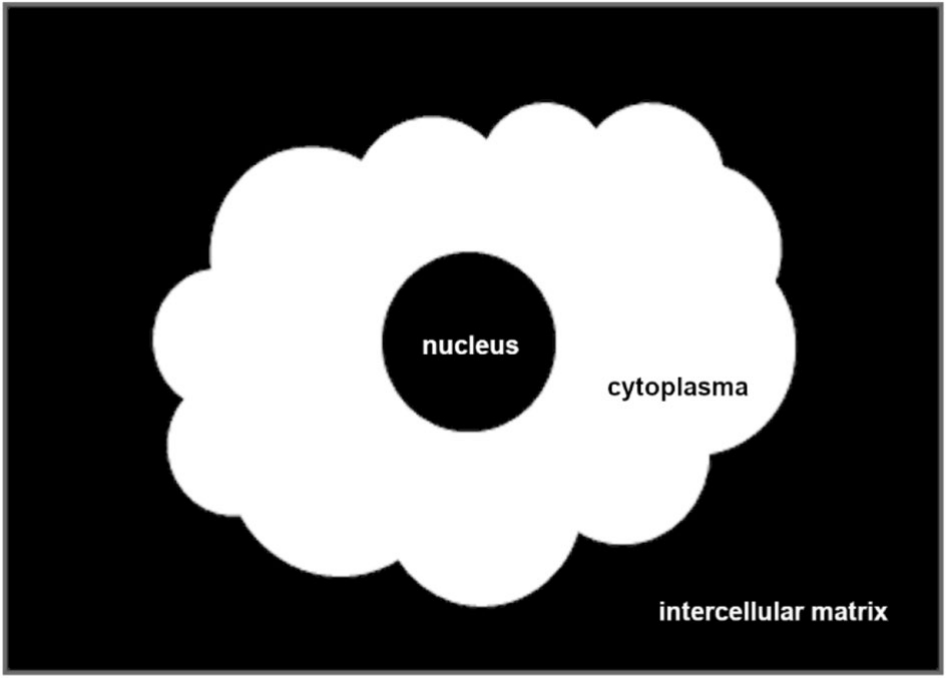
Cell model. The model for each cell contains a dark circular nucleus, a bright cell-cytoplasm of any shape, and a dark intercellular matrix.

The semantic assignment assigns the superpixel clusters generated by means of multi-stage merging to the object classes cell nuclei, cells (cytoplasm) and intercellular matrix. This classification is realized by evaluating appropriate features according to the model from Fig. 4. The simplified cell model includes nuclei with small intensity values. In contrast, for the cytoplasm, high intensity values with respect to the dynamic range of the data are assumed. While the cells may have any shape, the nucleus is typically circular. The nucleus contour is closed and completely surrounded by cytoplasm. The cytoplasm is also closed and completely surrounded by the intercellular matrix.

Based on the model, three uncorrelated features can be determined for nuclei detection. These include the shape, the appearance, and a segment relationship. The derivation of the cell cytoplasm and the intercellular matrix is based on a heuristic approach.

The shape of the nucleus is evaluated according to its *compactness*

$$C = \frac{{P^{2} }}{{4\pi \cdot A}}.$$

The compactness of a circle is 1 and increases as the perimeter $P$ of a segment increases in proportion to its area *A*.

As a characteristic for the appearance of a cell nucleus, the local *contrast* is used. This is described by the mean of the gradient magnitude $\left| {\nabla I} \right|~$ along the cell nucleus contour

$$\left| {\nabla I} \right| = \sqrt {\left( {\partial _{u} I\left( {u,v} \right)} \right)^{2} + \left( {\partial _{v} I\left( {u,v} \right)} \right)^{2} } ~,$$

where $\partial _{u} I\left( {u,v} \right)$ and $\partial _{v} I\left( {u,v} \right)$ represent the partial derivatives $\partial _{u}$ and $\partial _{v}$ of the two-dimensional image $I$ at the location $\left( {u,v} \right)$ in the directions $u$ and $v$, respectively.

The segment *relationship* takes the presence of an outer bright cell segment around a fully enclosed dark cell nucleus segment into account. If the partial derivatives are approximated by difference quotients,

$$I_{u}^{'} \left( {u,v} \right)\tilde{ = }\frac{{I\left( {u,v} \right) - I\left( {u - 1,v} \right)}}{{u - \left( {u - 1} \right)}} = I\left( {u,v} \right) - I\left( {u - 1,v} \right)$$

and

$$I_{v}^{'} \left( {u,v} \right)\tilde{ = }I\left( {u,v} \right) - I\left( {u,v - 1} \right),$$

and if the quotients are consistently assigned to the two segments, then the assignment of the outer cell–inner nucleus relationship (OCIN) can be made depending on the sign:

$$OCIN = \left\{ {\begin{array}{*{20}l} {nucleus,~} \hfill & {if~\mathop \sum \limits_{{k = 1}}^{m} \left( {I_{{inner_{k} }} - I_{{outer_{k} }} } \right) < 0;} \hfill \\ {cell,} \hfill & {otherwise;~} \hfill \\ \end{array} } \right.$$

where $I_{{inner_{k} }}$ denotes the intensity of contour pixel $k$ from the inner segment, $I_{{outer_{k} }}$ denotes the intensity of the outer segment pixel adjacent to the inner contour pixel $k$, and $m$ is the contour length of each inner segment contour. Ideally, any dark cell nucleus represented by $I_{{inner_{k} }}$ is classified as such if the surrounding tissue corresponds to a brighter cell represented by $I_{{outer_{k} }}$.

Considering the original data, the following additions to the model become necessary. Due to the high variance of the intercellular space, it is still characterized by many segments after the multi-stage merging step. Moreover, the cells do not always appear completely closed. Thus, the cell nuclei are partially adjacent to the segments of the intercellular matrix. Consequently, an algorithm is devised that assesses the intensity structure in a local region with the help of the OCIN parameter.

The algorithm reconstructs a complete hierarchy of nested contours on each image. A parent–child relationship occurs only when one segment is completely enclosed by another segment. For these segments, the OCIN parameter can be determined directly in the first iteration ($OCIN_{{min}} = 1$). If the inner segment is darker than the outer segment, the label nucleus is assigned.

**Figure 5:**
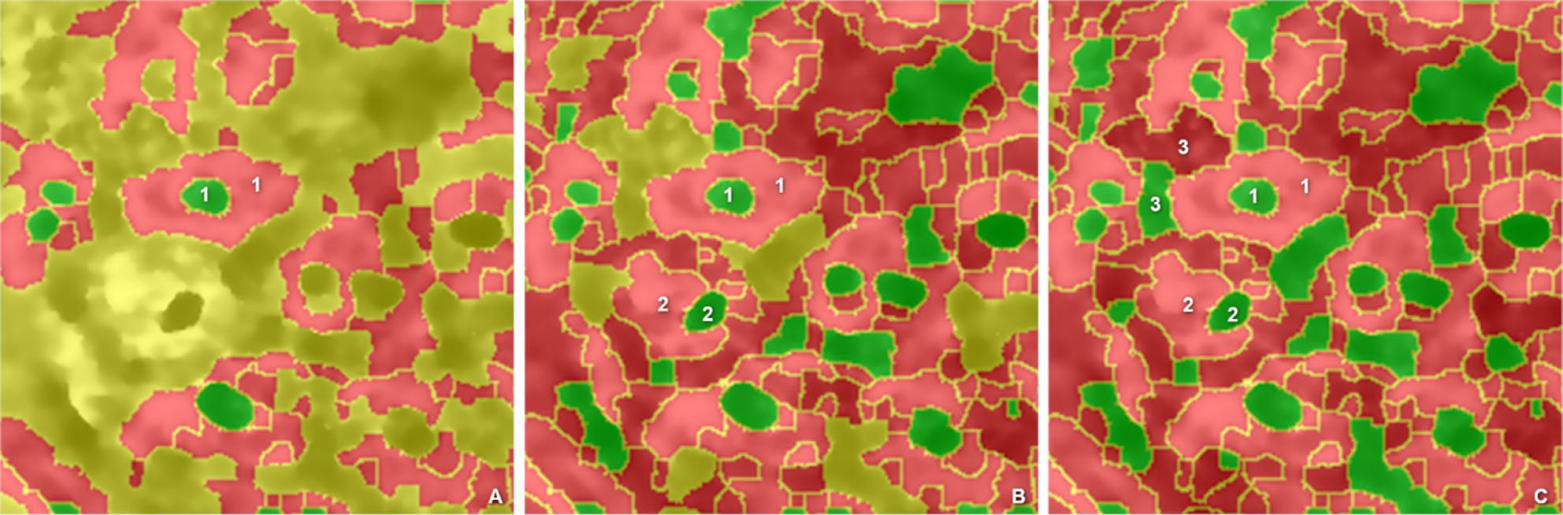
Classifications of nuclei. Potential nuclei (green), potential cells (red), and the residual set (yellow) for the first (**A**), second (**B**), and third (**C**) steps of the OCIN calculation. The segment-specific OCIN hierarchy index is 1 for all segments where a nucleus is fully enclosed by a cell in the first step (**A**), 2 for all segments where a nucleus is fully enclosed by a cell in the second step (**B**), and so on. An example for an OCIN hierarchy index in each step is indicated by the numbers in (**A**–**C**).

All segments that do not correspond to a parent–child relationship in the first iteration are defined as the residual set (Fig. 5A, yellow). The residual includes cells, nuclei, and segments of the intercellular matrix, which are iteratively reclassified in further steps. During these iterations, the differences between the neighboring contour boundaries are only calculated for the residual segments. This implies that the definition of a valid parent–child relationship is adjusted so that a completely enclosing parent segment is no longer required. Child segments can now additionally be considered valid and thus labeled if they are adjacent to multiple parent segments. The condition is that only one of the parent segments has not yet received an OCIN label. The parent segments already labeled in the previous iterations are no longer considered for the current stage. This modeling of a 1:1 parent–child situation follows the described rules of a single fully enclosed inner segment. All segments in each reclassification step with a positive sign are labelled as potential cell segments. The remaining distinct segments with a negative sign are assigned to the class of potential nuclei. Adjacent segments with negative signs remain in the residual set. The reclassification is performed until all segments have been assigned to the cell or nucleus class. In the end, each segment is labelled as a potential nucleus or as a potential cell (Fig. 5C). A delimitation of the intercellular matrix is not necessary for this step. The $OCIN_{{max}}$ parameter indicates the last iteration that is required to completely assign the OCIN parameter to all segments. The parameter named *OCIN hierarchy index* $H$ represents the step of iteration $i$ with $i \in \left[ {OCIN_{{min}} ,OCIN_{{max}} } \right]$ in which this OCIN relationship has been set for a specific segment:

$$H = i,~\quad if\;OCIN\left( i \right) \leftarrow cell|nucleus$$

**Figure 6:**
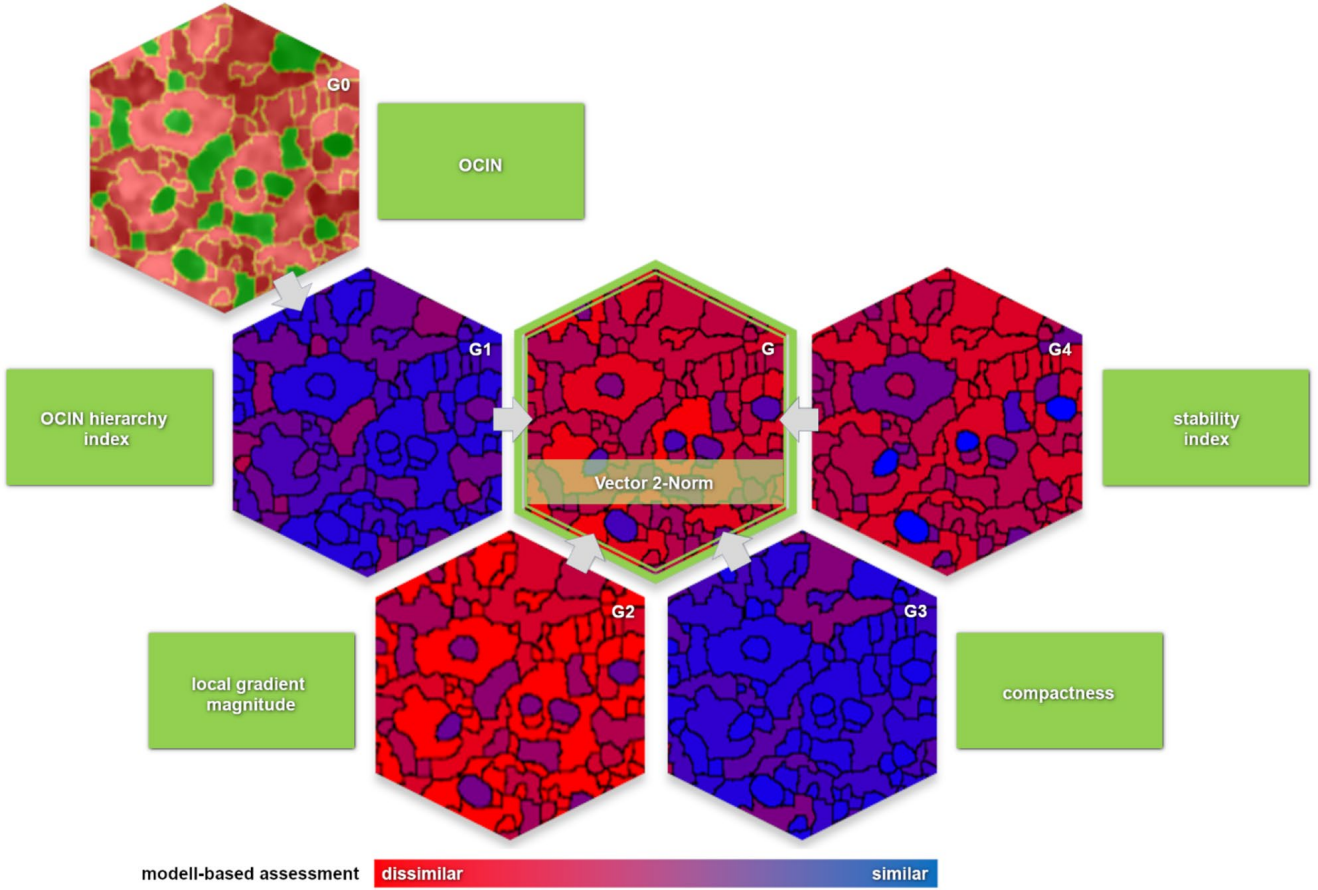
Visualization of the five extracted suitable base features. Outer cell–inner nucleus relationship “OCIN” (**G0**), potential cytoplasm (red), potential nuclei (green) as the basis for cytoplasm–nucleus classification and for determination of OCIN hierarchy (**G1**), local gradient magnitude (**G2**), compactness (**G3**), and stability index (**G4**) derived by module of feature extraction (Fig. 2G) and the resulting image by vector 2-norm (**G**) as measure of similarity to the nucleus-model (dissimilar (red), similar (blue)).

In summary, a relationship is set once a dark segment is completely surrounded by brighter segments. This consideration leads to the classification of dark segments as nuclei. In the process, individual dark segments of the intercellular spaces will also be labelled as nuclei (Fig. 6 G0). To separate the misclassifications, a fuzzy-like feature evaluation is performed.

The features stability index $S$ resulting from multi-stage merging (Fig. 6 G4), compactness $C$ (Fig. 6 G3), local gradient magnitude $~\left| {\nabla {\text{I}}} \right|$ (Fig. 6 G2), and OCIN hierarchy index $H$ (Fig. 6 G1) are used for this purpose. The probability that a segment corresponds to a nucleus increases as the segment features become more similar to the model. This similarity $d$ is determined by the Euclidean norm between the current segment feature vector $\vec{v}$ and the reference vector $\overrightarrow {{ref}} = \left( {MSM_{{max}} ,I_{{max}} ,~C_{{min}} ,~OCIN_{{min}} } \right)^{T}$, where $MSM_{{max}}$ is the maximum number of merging steps (given by the number of thresholds $T_{m}$, here 7), $I_{{max}}$ is the maximum value of intensity (given by the data type of image, here 255), $C_{{min}}$ is $1.0$ for compactness of the circle, and $OCIN_{{min}}$ is $1.0$ for detection of a significant cell–nucleus relationship in the first iteration.

To obtain an equally weighted influence of the features for the similarity evaluation, the feature vectors are normalized. The maxima for $C_{{max}}$ and $OCIN_{{max}}$ are taken from the calculated data to get normalized values for $C$ and $H$ within the range $\left( {\left. {0,1} \right]} \right.$. The result is a scalar $d_{i}$ as a measure of similarity to the model and is referred to below as the quality value of a nucleus (Figs. 2G, 6G).

$$\begin{aligned} d_{i} \left( {\left\| {\overrightarrow {{ref}} } \right\|,\left\| {\vec{v}} \right\|} \right) & = \left\| {\left\| {\vec{v}_{i} } \right\| - \left\| {\overrightarrow {{ref}} _{2} } \right\|} \right\| \\ & = \left\| {\left( {\begin{array}{*{20}c} {S_{i} {\text{/}}MSM_{{\max }} } \\ {\left| {\nabla I} \right|_{i} {\text{/}}I_{{\max }} } \\ {\left( {C_{{\max }} + 1 - C_{i} } \right){\text{/}}C_{{\max }} } \\ {\left( {OCIN_{{\max }} + 1 - H_{i} } \right){\text{/}}OCIN_{{\max }} } \\ \end{array} } \right) - \left( {\begin{array}{*{20}c} 1 \\ 1 \\ {\begin{array}{*{20}c} 1 \\ 1 \\ \end{array} } \\ \end{array} } \right)} \right\|_{2} ,~~~~~ \\ \end{aligned}$$

with $i = 1..n$ and n is the number of overall segments in $I_{{MSM}}$. Increasing values of $d_{i}$ are related to the decreasing similarity to the nucleus model and thus a smaller quality.

The selection of the most significant nuclei is realized by a relative quality threshold $T_{{qual}}$. This threshold value represents the permissible relative minimum of quality as a function of the calculated quality values $d$

$$Label_{i}= \left\{{\begin{array}{*{20}l}{nucleus,~}& {if \; d_{i} < {(max(d)}-{min(d))}*\ (1-T_{qual})+min(d);} \\ {0,}&  {otherwise;~} \\ \end{array} } \right. $$.

A segment is finally labelled as a nucleus if the quality of the pre-classified nucleus segment exceeds the resulting absolute threshold. The scene-based derivation of a suitable threshold $T_{{qual}}$ with the help of the comparison of the segments from multi-stage merging and the ground truth using the Dice coefficient is explained below.

Since the classification of nuclei is based on the OCIN relationship and the segments labelled as nuclei have been assigned a quality value, for the most significant nuclei, the adjacent segments can be marked as surrounding cells (Fig. 2H). Any remaining regions not labelled as nucleus or cell are automatically assigned to the intercellular matrix.

## Results

**Figure 7:**
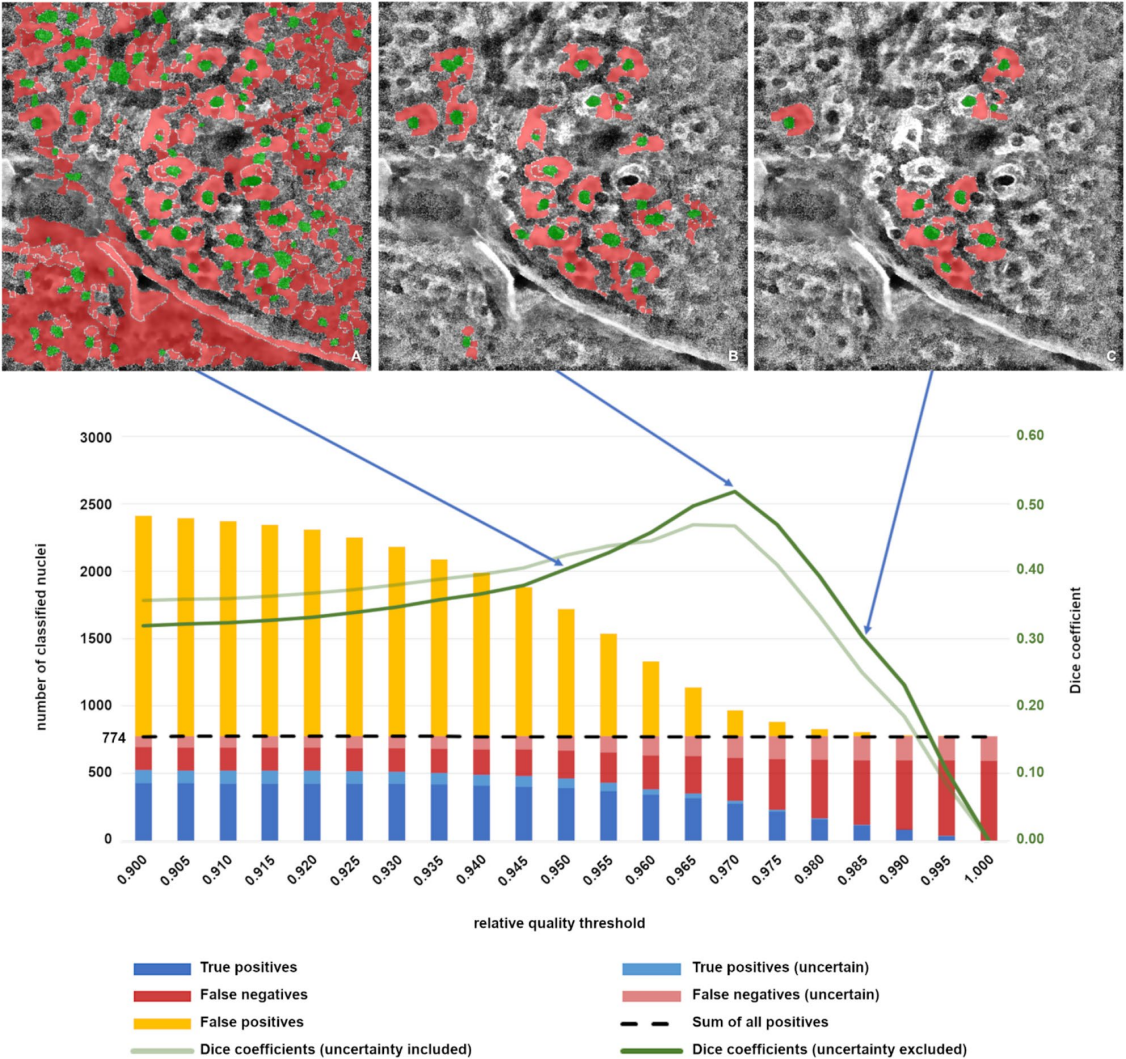
Classification results. (**A**–**C**) Overlay of cell cytoplasm (red) and nuclei (green) to slice 31 using relative quality thresholds (blue arrows) with value 0.95 (**A**), 0.97 (**B**), and 0.985 (**C**), (**D**) Summation of all true positives and misclassifications (left ordinate) for Slices 29–40, 45, 50, and Dice coefficient (right ordinate) with maximum at threshold 0.97 using different relative quality thresholds (abscissa). The number of total nuclei from ground truth is 774 (including certain and uncertain nuclei).

Figure 7 presents an overlay to the cell and nucleus regions on the multiphoton data for one slice (A–C) as well as the assignment of the quality value $d_{i}$ for each segment for all slices (D). The quality value is only defined for cell nucleus segments according to the considered model. For this reason, the evaluation (Fig. 7D) is limited to cell nuclei.

Figure 7D also shows the number of correct classified nuclei (true positives) and the misclassifications. Misclassification includes both false negatives and false positives. These classifications are subdivided into certain or uncertain class affiliations due to the data properties according to the ground truth labelling.

The ground truth data correspond to the image stack introduced above with 774 nuclei within the 14 layers of a single image stack. The number of nuclei indicated on the left ordinate corresponds to the final detected nuclei with respect to the relative quality threshold $T_{{qual}}$ within these 14 layers. The abscissa indicates the applied relational threshold $T_{{qual}}$. As the threshold increases, only the most significant nuclei due to the high-quality values are designated (Fig. 7 A–C). The number of false positives decreases with increasing quality threshold. However, the number of true positives also decreases.

The graphs for the Dice coefficients in Fig. 7D can be used to derive the optimal value for $T_{{qual}}$. From the 21 relative thresholds given on the x-axis, the most suitable one is given by the maximum of the Dice coefficient. Both functions have their maximum at the relative quality threshold of 0.97. Thus, this result is independent of the use or non-use of the uncertain segments. The determined value represents the best global result in terms of the true positive and false positive ratio considering the whole image stack.

**Figure 8:**
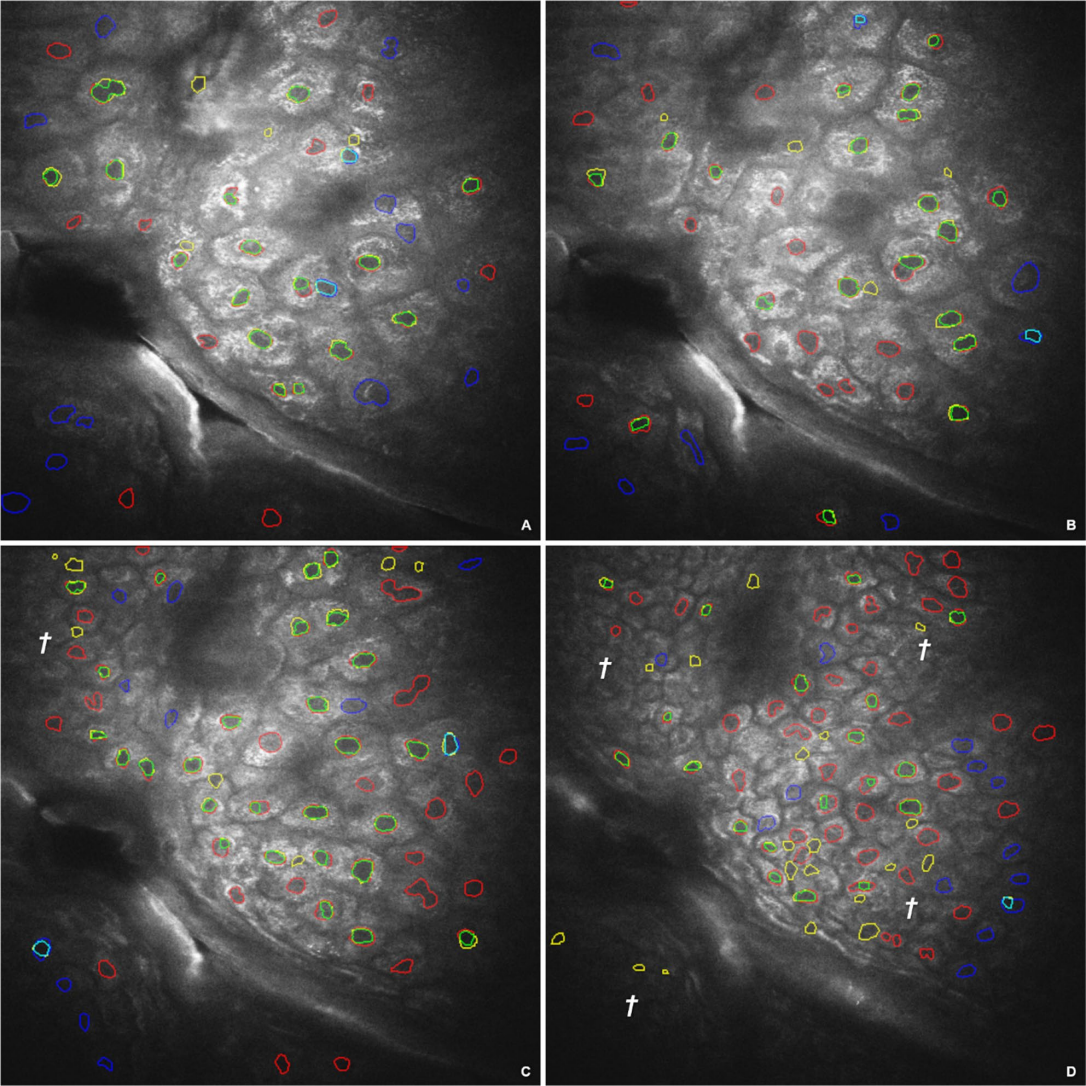
Ground truth comparison. Ground truth (GT) and automatic segmentation using multi-stage merging and relative quality threshold 0.97 on original input image of Slice 30 (**A**), 33 (**B**), 37 (**C**), and 45 (**D**). Red contours correspond to the certain nuclei of GT, blue contours correspond to the uncertain ones, green contours correspond to the segmented regions that coincide with the certain GT segments, cyan contours correspond to the segmented uncertain GT contours, and yellow outlines are false positives. † marks peripheral areas with ambiguous cell structures.

Figure 8 illustrates the nuclei of the ground truth and the nuclei extracted by multi-stage merging with subsequent semantic assignment for the layers 30, 33, 37, and 45. Table 1 shows the absolute values in addition to the slice images from Fig. 8 and the other images of ground truth.

**Table 1:** Comparison of true positives and misclassifications.

Slice	Threshold	True positive (TP)	TP uncertain	False positive	False negative (FN)	FN uncertain
29	0.970	11	1	6	12	15
	0.955†	13	8	13	10	8
	0.960*	13	3	8	10	13
30	0.970†	16	2	4	10	12
	0.965*	19	2	9	7	12
31	0.970*†	**17**	**1**	**5**	**8**	**6**
32	0.970	11	1	2	16	5
	0.960*†	16	1	6	11	5
33	0.970*†	19	2	4	14	6
34	0.970	17	1	4	31	9
	0.945*†	34	6	30	14	4
35	0.970*†	25	2	14	14	10
36	0.970†	27	2	20	8	11
	0.980*	19	0	2	16	13
37	0.970*†	27	2	7	22	9
38	0.970	10	0	1	44	23
	0.945†	31	5	26	23	19
	0.950*	28	2	15	26	21
39	0.970*	25	1	13	30	19
	0.965†	32	6	33	23	14
40	0.970	31	1	24	27	20
	0.965*†	38	3	42	20	18
45	0.970*†	19	1	19	34	12
50	0.970†	20	2	69	49	4
	0.975*	17	1	47	52	5

The comparison uses the global relative quality threshold 0.97 and the slice specific best threshold (indicated by * without uncertainty and by † with uncertainty) given by the maximum of the dice coefficient. The overall maximum of the dice coefficient (bold) is given for slice 31 using the threshold 0.970.

The optimal quality thresholds for the individual layers are compared to the threshold derived from the entire image stack. While the global threshold is identical to the threshold specific to layers 31, 33, 35, 37 and 45, there are some deviations for the other layers. The best segmentation rated by the dice coefficient is given for slice 31 $DC~ = ~0.65$ ($0.72$, *excluding the uncertain segments). Based on the calculation of how many pixels were correctly classified with respect to the binary decision to be a nucleus or non-nucleus, this results in a value of 97.8%. This means that only 2.2% of the total image pixels in slice 31 were misclassified with regard to the classification as a cell nucleus. With increasing layer depth, the classification rate decreases from $DC~ = ~0.60$ ($0.70$*) in layer 30 to $DC~ = ~0.60$ ($0.65$*) in layer 37 and further to $DC~ = ~0.38$ ($0.41$*) in layer 45.

**Figure 9:**
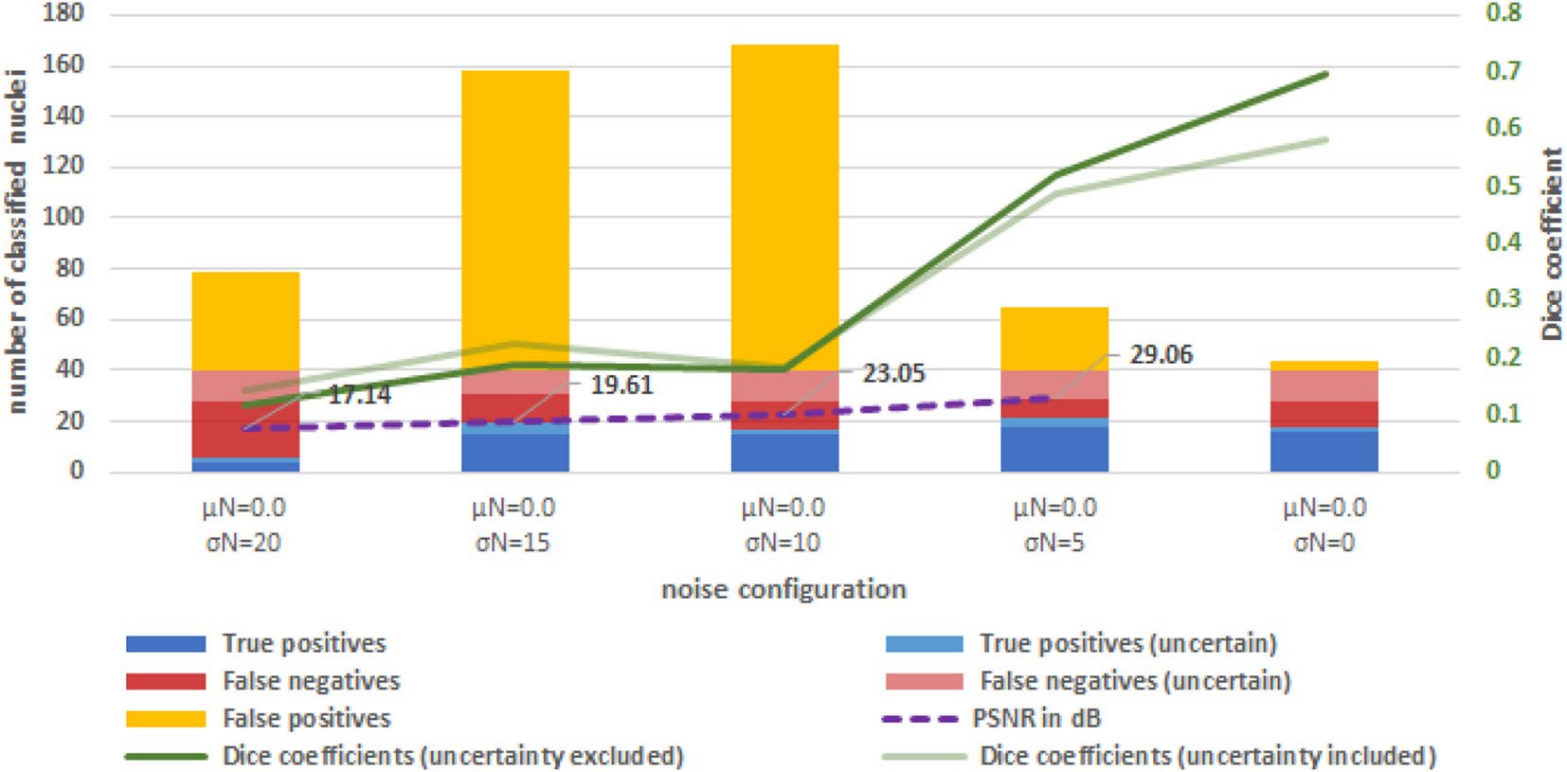
Classification results for noisy images. Summation of all true positives and misclassifications (left ordinate) for slice 30, PSNR in respect to image with $\mu _{N} = 0$ and $\sigma _{N} = 0.0$ and Dice coefficient (right ordinate). The number of total nuclei from ground truth is 40 (including certain and uncertain nuclei).

To address the limitation of quality assessment based on the evaluation of a single image stack, an additional noise process was modeled (Fig. 9). The image quality of the original unfiltered image $I$ of layer 30 with defined noise mean value $\mu _{N} = 0$ and noise standard deviation $\sigma _{N} = 0.0$ was modified by adding Gaussian noise with fixed $\mu _{N} = 0$ and variable $\sigma _{N} = i \cdot 5.0$ with $i = 1..4$. This results in a PSNR of $29.06\;{\text{dB}},\;23.05\;{\text{dB}},~\;19.61\;{\text{dB}}$, and $17.14~\;{\text{dB}}$ with respect to $I$. Comparable Dice coefficients between $DC = \left[ {0.49,~0.58} \right]$ and [$0.52,~0.69]$ (excluding the uncertain segments) are obtained for the threshold $T_{{qual}} ~ = ~0.97$ for the modified images with a $\sigma _{N} \le 5$. The Dice coefficient decreases to below $DC = 0.14$ with increasing noise, mainly due to excessive plateauing caused by the top-hat transformation.

The recognizability of peripheral cell structures decreases with increasing layer depth. Reasons for this are the decrease in cell size and contrast. Clear structural boundaries are no longer perceptible. Figure 8D and parts of Fig. 8C also show areas that could not be reconstructed sufficiently well by experts to make a reliable statement about the cell and nucleus boundary. Many of the algorithmic misclassifications are in such structures.

**Figure 10:**
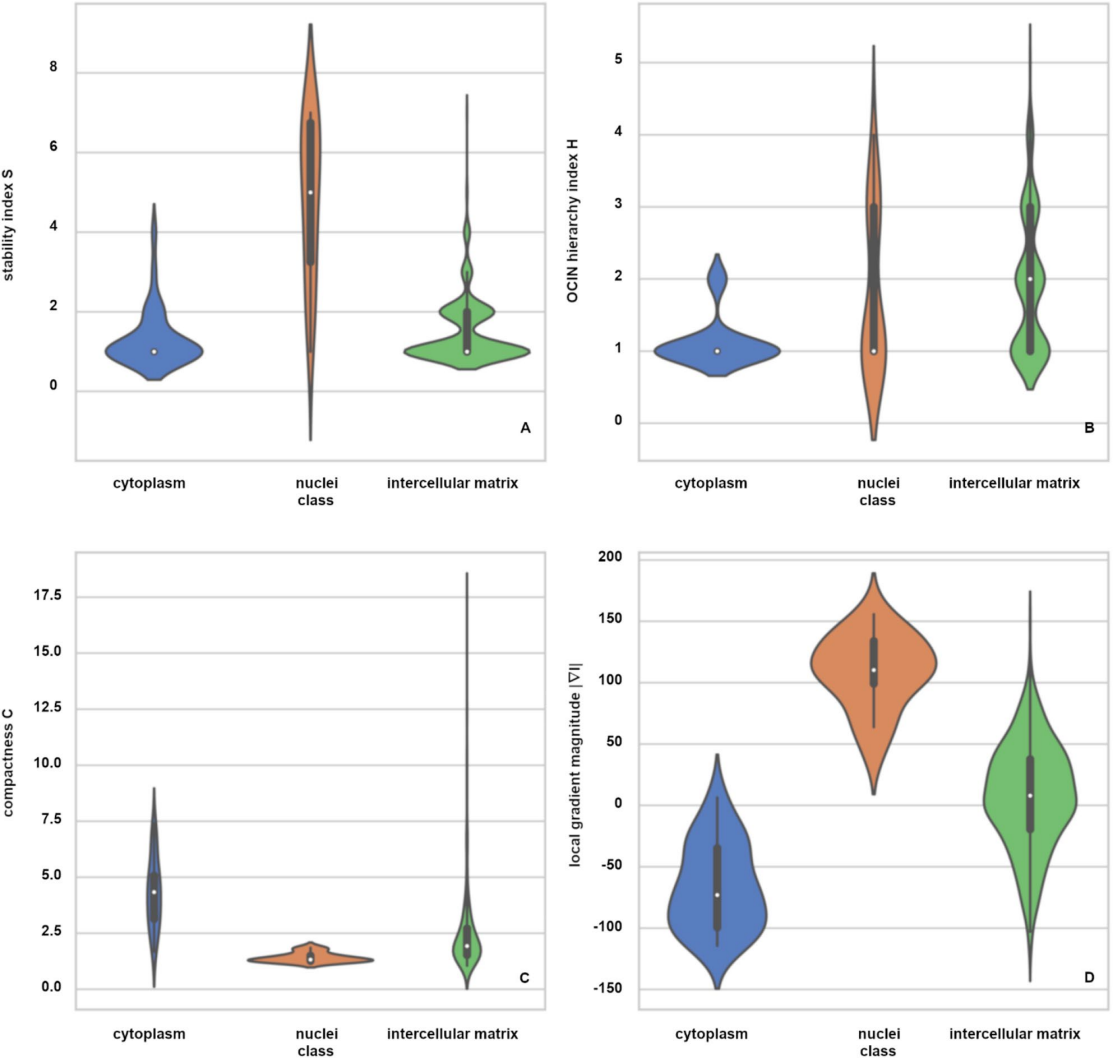
Feature distributions. Violin plots for the features stability index (**A**), OCIN hierarchy index (**B**), compactness (**C**), and local gradient magnitude (**D**) separated according to the classes cytoplasm (blue), nuclei (orange), and intercellular matrix (green) for slice 31 with visualization of median (white dot), interquartile range (IQR, bold black bar), first quartile—1.5 IQR, and third quartile + 1.5 IQR, respectively (thin black lines) and outlier (without any lines).

The distribution of the used features is represented by violin plots for cytoplasm, nuclei, and an intercellular matrix (Fig. 10). The class affiliation is given by the ground truth, whereby the features of uncertain segments are also included. A registration of the segments from the multi-stage merging approach and segments from the ground truth was deployed to evaluate the distributions of the features for the aforementioned classes. The homologous segments of our multi-stage merging approach are determined for each cytoplasm and nucleus segment of the ground truth based on the maximization of the common area coverage. Only the individual nucleus segments with a segment-specific $DC \ge 0.75$ are used for the visualization in the violin plots. However, all single segments that are located in the area of the intercellular matrix of ground truth are considered.

Figure 10A shows the statistics of the stability index. The cytoplasm and the intercellular matrix classes are characterized by a lower level of stability than the nuclei. The stability index for the intercellular matrix scatters slightly more than the one for the cytoplasm, while the scatter is highest for the nuclei. For the OCIN index (Fig. 10B), we observe a difference in the median value for the intercellular matrix ($\tilde{H} = 2$) versus the other two classes ($\tilde{H} = 1$). However, the spread of the distribution is very similar for the nuclei and the intercellular matrix. The violin plots of compactness (Fig. 10C) show the almost circular shape of nuclei in contrast to the wide scattering of the cytoplasm and intercellular segments. However, the intercellular space, in particular, contains many small segments of high compactness. The plot of the local gradient magnitude (Fig. 10D) illustrates the positive sign for all cell nucleus segments. These are darker than their environment due to a lack of fluorescents. The contrast of the cytoplasm is brighter, but also darker relative to their environment. This is due to the adjacent nuclei on the one hand, and to the adjacent bright structures in the intercellular matrix on the other hand. The intercellular matrix has a nearly symmetrical distribution. This is partly due to the surrounding cytoplasm, but also the occurrence of many small segments with high intensity variance in the intercellular space itself.

None of the four features depicted in Fig. 10 significantly differentiates the classes from each other. Only the combination of the features leads to the separation of the nuclei.

## Discussion

Our proposed multi-stage merging with subsequent combination of several weak features enables the automatic classification of the nuclei and adjacent cell regions. Cells are detected only due to the presence of significant internal nuclei. Unlike many other approaches, no additional modalities (e.g., RCM, FRET, FLIM) are required. Moreover, our method is intended to contribute to the automatic scale-independent, and thus layer-independent, segmentation of skin cells.

The evaluation of the processing pipeline requires the consideration of the anatomically induced variable data characteristic resulting from the MPT. In particular, the intercellular spaces and contrasts, which become smaller as the layer depth increases, and the irregularity of the cell boundaries are a challenge. The irregularity affects the shape, which varies, as well as the size of the cells and nuclei. The cytoplasm provides isolated fluorescence in cell regions. A uniform intensity distribution or larger homogeneities do not occur. For this reason, a scale-independent methodology has been developed which dispenses with the use of an "optimal" parameterization.

A segmentation of human cells was also carried out by Walsh and Skala^15^ and, in particular for human skin, by Decencière et al.^16^ and Chen et al.^19^. While Walsh and Skala^15^ used a direct parameter for limiting the permissible cell size (only diameters between 6 and 25 pixels were approved) this corresponds to 2.34 μm–9.77 μm considering our data resolution), in Decencière et al.^16^, this size was restricted by a morphological horizontal area with a given size of *A* = 150 μm^2^ as the cell size minimum. An exemplary dimension of 29.5 × 16.9 μm is given for the bounding box of the marked cell in our Fig. 1B at a depth of 31 μm. Due to this lateral size of skin cells of the stratum spinosum, the approaches proposed in^15,16^ are less suitable to segment these skin layers in the available MPT data without further adjustments.

In a publication by Chen et al.^19^, the cell size was only indirectly limited by the radius of the Gaussian kernel to smooth the gradient magnitude image before the watershed transformation. However, this approach is based on a segmentation of the cell contours in the additional RCM data. These segments were necessary for the initialization of the algorithms for the detection of nuclei and cytoplasm in the MPT data.

The use of a scale-independent algorithm has a decisive disadvantage: instead of optimally tuning the algorithm to a layer depth, a configuration is used that acquires small details. This is also necessary because different cell types and, thus, sizes can occur in a single-layer depth due to the skin anatomy. The methods of pre-processing are mainly affected by different scales. These methods are all dependent on kernel size. For the kernels of the top hat transformation (11 × 11 pixels), the histogram equalization (16 × 16 pixel), and the anisotropic filtering (3 × 3 pixels), a compromise between filter effect and preservation of small structures was chosen. The top hat transform creates larger contiguous plateaus with only minimal disruption of the intercellular spaces. The histogram equalization increases the contrast but also the variance of the intensities in the previously created homogeneous plateaus. For this reason, an edge-preserving filtering according to the principle of anisotropic diffusion was used. The noise was effectively minimized with adequate plateau formation for the subsequent steps. However, due to these pre-processing steps, signal drop-induced artefacts occurred in the periphery. These artefacts can be ignored because there are no relevant structures to be analyzed at the image periphery.

The watershed transformation realizes an edge-based over-segmentation rather than extracting coherent segments with specific intensity distributions. This over-segmentation results in the division of the semantic objects. The degree of over-segmentation can be restricted by a previous Gauss-smoothing at the edge image. We propose a multi-stage merging approach for the reliable reduction of over-segmentation.

Merging multiple parts of a single semantic object into the coherent object ensures the extraction of typical object features, such as shape and topology. Using a single, possibly non-optimal, threshold or segmentation algorithm with too many constraints results in less accurate results. The use of multiple thresholds $T_{m}$ with subsequent merging allows a robust segmentation of existing tissue situations. Seven thresholds are sufficient for our application because only a small number of gradients of adjacent segments exceed the selected maximum threshold, $T_{m} = 70$. The other thresholds realize an equidistant coverage of the range of gradient magnitudes down to $T_{m} = 10$. In our application, a gradation of the threshold in steps of 10 turned out to be sufficiently precise with adequate performance. A reduction in the step size between the individual thresholds increases the dynamic range of the OCIN hierarchy and thus increases its resolution, but it also increases the effort of multi-stage merging. However, this ultimately might not result in a significant change in the segmentation. Alternatively, threshold values can be used that realize an equidistant coverage of the range of values defined by the data material. For example, up to 256 threshold values can be used for data with a dynamic range of 8 bits. This is particularly useful if there is no knowledge of the semantic data properties.

The reduction of the number of segments by using our developed multi-stage merging leads to a decrease in computing time of the subsequent steps. In addition, the process of multi-stage merging results in a novel quality criterion called stability index. Three additional features are determined in addition to the stability index: the compactness, the local gradient magnitude, and the novel OCIN hierarchy index. A combination of these features is used since the individual features do not allow an obvious demarcation of cells, cell nuclei, and intercellular matrix.

Other features were discarded after they were examined. In addition to the local gradient magnitude based on the contour pixels, the global gradient magnitude was determined based on the mean intensities of adjacent segments, which turned out to be less adaptive. Shape characteristics, such as size of areas and diameters, were in principle unsuitable due to the data properties. The feature of compactness has been given priority over the eccentricity parameter. The appearance features of the objects have all proven insufficient for a classification: mean intensity, variance, minimum, median, and maximum intensity. Textural features were also examined to delineate the cells in the raw data without morphological pre-processing. The fluorophore-containing cell regions show a pattern of higher intensities in contrast to the regions of the nuclei and the intercellular matrix. However, a suitability of the determined Haralick texture features^48^ for cell detection could not be demonstrated.

The problem of the similarity of nuclei and the intercellular matrix generally consists in their low-textured appearances. The apparent separation by their shape or size is not possible due to the over-segmentation in areas of the intercellular matrix caused by the large intensity variability. These intercellular matrix areas remain largely over-segmented even after multi-stage merging. The advantage of the restrictive merging character is that the nuclei can be separated despite direct contact to the intercellular matrix. Misclassified intercellular matrix segments can be discarded by the combination of the features mentioned above. The most significant potential nucleus segments that meet the quality threshold, $T_{{qual}}$, are labelled as nuclei. The quality threshold, $T_{{qual}} = 0.97$, was determined using the maximum of the Dice coefficients based on the automatic segmentation and the ground truth of the entire image stack. There is a slight deviation between this global optimum and the threshold value determined by the specific layer. Considering the layer-specific maxima of the $DC$ and their associated thresholds, the median for $T_{{qual}}$ is also 0.97.

A limitation results from our basic approach of the extraction of cell parts. The cell cytoplasm is extracted only in dependence of the evaluated nuclei. A separate quality evaluation of cytoplasm segments and their detection in case of an absence of nuclei remains to be investigated. As a second limitation, ambiguities are possibly caused by the over-segmentation resulting from the large intensity variability in areas of the intercellular matrix in combination with the less textured appearance of the nuclei. These ambiguities can only be resolved to a limited extent by semantic assignment. Thus, the choice of the threshold $T_{{qual}}$ represents a compromise between the number of true positives and false negatives.

Another limitation consists in the different annotations including different levels of uncertainty provided by the two experts. This discrepancy is important information, as it gives an idea of the difficulty of the segmentation as well as a reference when evaluating the error of the proposed method. In clinical practice, typically only one expert will look at the images. Consequently, a study on the spread of the annotation results with several experts including a comparison of ways to reach consensus among the experts would be valuable.

Other approaches to reduce false positives include the exclusivity test, where adjacent cell segments can only be assigned to one nucleus. This procedure was evaluated as unsuitable due to the partly unrecognizable cell boundaries and the occurrence of multiple cell nuclei in single cells. Another approach was to include adjacent layers. If there is a correspondence for a detected cell nucleus in the adjacent layers, then this cell nucleus classification can be regarded as reliable. In particular, the axial resolution is the limiting parameter for the realization of a cross-layer processing. Even the supposed high axial resolution of 1 μm does not meet the requirement of depth-graded evaluation.

In addition to the exclusive use of TPEF MPT data, alternative modalities, such as SHG^5^ and FLIM^6–8^, will be considered in future work. The integration of further information will likely increase the reliability of the segmentation by reducing the uncertainties that result from a single modality. For example, FLIM imaging expands the dynamic range of the fluorescence areas, thus the distinction between cell areas and other structures will be improved.

The feasibility of applying the presented technique in clinical practice requires discussion of two domains. The first domain involves the examination and associated data acquisition on the patient by multiphoton tomography. A depth scan is performed for a skin region of diagnostic relevance. The resulting image stack contains multiple depth-staggered slice images, depending on the requirements and the scanning regime set, where scanning a single slice with a resolution of 200 × 200 μm^2^ takes about 6.5 s. Our proposed algorithm is based on processing single images. The robustness of a future algorithmic tissue type classification for diagnostic support might be increased by considering multiple slices.

The second domain involves the analysis of the data material by our proposed algorithm. This is done offline in the prototypical phase so far. It does not require much expertise by the user, as manual parameterization is not required. Only the restriction to the most significant cells may require manual readjustment of the final threshold $T_{{qual}}$, as discussed earlier. The hardware costs for the image processing system are low since non-proprietary consumer hardware is sufficient for the application. The performance mainly depends on the CPU or GPU. The segmentation currently takes about 32.1 s per slice image on a system with Intel® Core™ i7-9850H CPU @ 2.60 GHz and 48 GB RAM using all 6 cores. Especially the presented multi-stage merging benefits from the possibility of parallel processing. A further performance increase is possible by transferring the algorithms to a GPU. Processing of the single images and examination of the segmentation results would be possible during tomography. The segmentation forms a basis for the diagnosis by the medical staff. The results could also influence the diagnostic process so that, for example, its termination can be initiated if the data situation and segmentation quality are sufficiently good.

Since the field of application of our algorithm is the segmentation of anatomical structures of the human skin, a future use for the detection of different disease patterns, such as malignant melanoma but also NMSCs, is conceivable.

## Conclusions

In this work, we proposed a novel scale-independent methodology to identify skin cells in multiphoton data. We provide a robust strategy that addresses the anatomy-induced variable data characteristics, such as the depth-dependent variable cell sizes and contrasts, the speckle-like fluorescence response characteristics of the cytoplasm, and the cell boundary irregularity. To detect even small cells of the deeper skin layers, a compromise between filter effect and preservation of small structures was chosen. While only the pre-processing module has a size reference regarding its kernel sizes, the modules for segmentation and semantic assignment are completely size independent.

In our segmentation module, we propose a multi-stage merging approach for merging multiple parts of a single semantic object into a coherent object. Multiple thresholds are used, which can be automatically derived without knowledge of the data content based on their global image dynamics. This results in the general usability of the algorithm for other image processing problems. The fully automated segmentation and classification of human skin cells by our image processing pipeline provides a basis for the future classification of healthy and diseased skin areas.

## Supplementary Information

Supplementary Information 1.

Supplementary Information 2.

Supplementary Information 3.

Supplementary Information 4.

Supplementary Information 5.

Supplementary Information 6.

Supplementary Information 7.

Supplementary Information 8.

## Data Availability

All datasets generated for this study are included in the article/Supplementary Material.
