# Supplementary material for: Automatic segmentation of skin cells in multiphoton data using multi-stage merging
Source: Sci Rep. 2021 Jul 15;11:14534. doi: 10.1038/s41598-021-93682-y (PMC8282875; doi:10.1038/s41598-021-93682-y)

Automatic Segmentation of Skin Cells in Multiphoton Data using Multi-Stage Merging

Philipp Prinke[1,*], Jens Haueisen[1], Sascha Klee[1,2], Muhammad Qurhanul Rizqie[1,3], Eko Supriyanto[1,4], Karsten König[5,6], Hans Georg Breunig[5,6], Łukasz Piątek[1,7]

[1] Institute for Biomedical Engineering and Informatics, Technische Universität Ilmenau, 98693 Ilmenau, Germany.
[2] Karl Landsteiner University of Health Sciences, Department of General Health Studies, Division Biostatistics and Data Science, Dr. Karl-Dorrek-Straße 30, 3500, Krems, Austria
[3] Informatics Engineering Program, Universitas Sriwijaya, Palembang, South Sumatera, Indonesia.
[4] IJN-UTM Cardiovascular Engineering Centre, Universiti Teknologi Malaysia | UTM, Johor Bahru, Johor, Malaysia.
[5] Department of Biophotonics and Laser Technology, Saarland University, Campus A5.1, 66123 Saarbrücken, Germany
[6] JenLab GmbH, Johann-Hittorf-Straße 8, 12489 Berlin, Germany
[7] Department of Artificial Intelligence, University of Information Technology and Management, H. Sucharskiego 2 Str., 35-225 Rzeszów, Poland.
[*]Corresponding author. email: philipp.prinke@tu-ilmenau.de

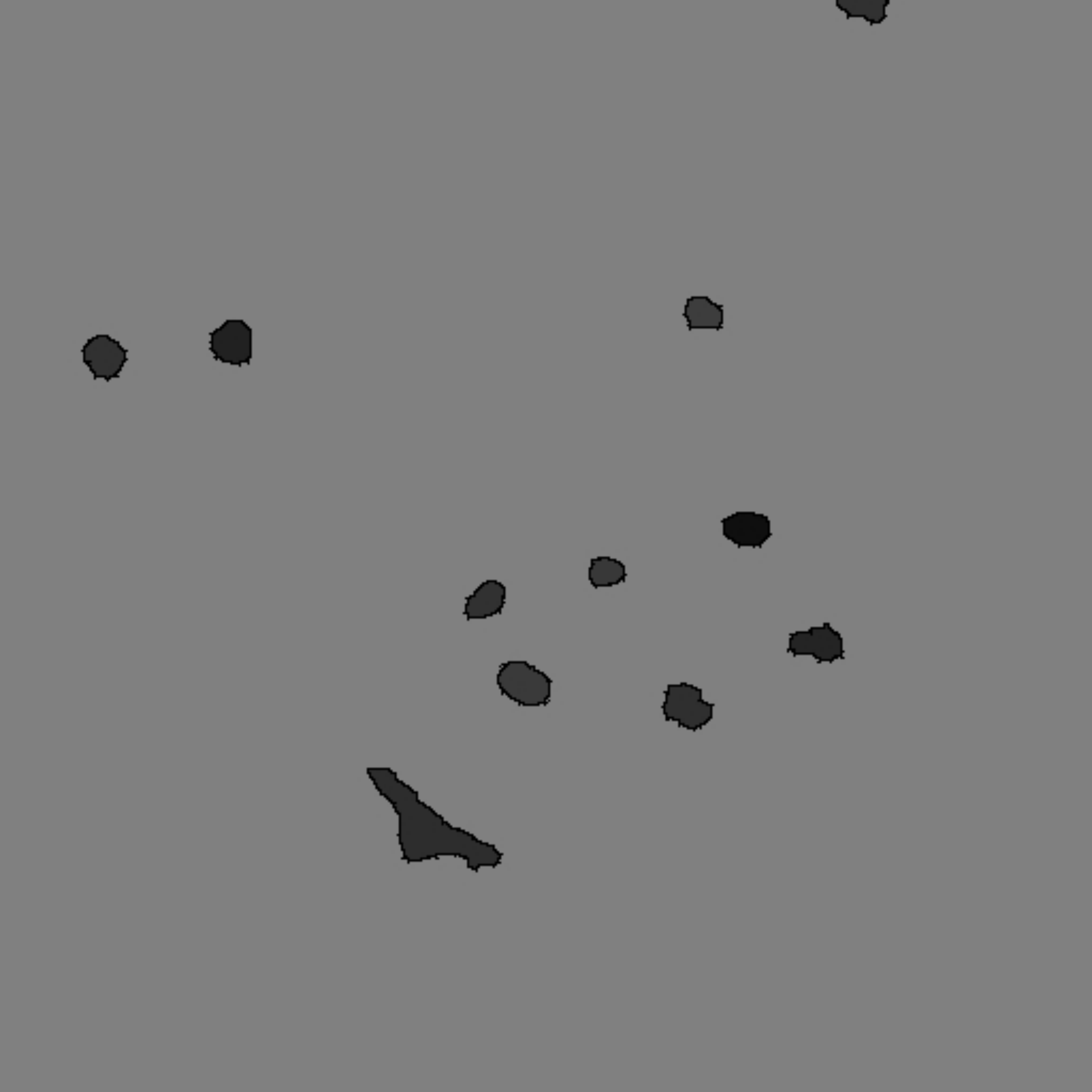

Supplement: Supplementary file 2 — Supplementary Information 2. [file 41598_2021_93682_MOESM2_ESM.pdf]
